# Enabling appropriate use of antibiotics: review of European Union procedures of harmonising product information, 2007 to 2020

**DOI:** 10.2807/1560-7917.ES.2020.25.45.2000035

**Published:** 2020-11-12

**Authors:** Aleksandra Opalska, Marcel Kwa, Hubert Leufkens, Helga Gardarsdottir

**Affiliations:** 1 Division Pharmacoepidemiology and Clinical Pharmacology, Utrecht Institute for Pharmaceutical Sciences, Faculty of Science, Utrecht University, Utrecht, the Netherlands; 2 Directorate-General for Health and Food Safety, European Commission, Brussels, Belgium; 3 Department of Pharmacovigilance, Medicines Evaluation Board, Utrecht, the Netherlands; 4 Department of Clinical Pharmacy, Division Laboratories, Pharmacy and Biomedical genetics, University Medical Center Utrecht, Utrecht, the Netherlands

**Keywords:** antimicrobial resistance, antibiotics, appropriate use of antibiotics, product information, European regulatory measures, referral procedures

## Abstract

**Introduction:**

Antimicrobial resistance (AMR) is one of the most important challenges in modern clinical practice. The European regulatory network has a strategy to support prevention of AMR by applying specific referral procedures.

**Aim:**

The aim of this study was to evaluate post-authorisation changes made in the product information of key antibiotics that underwent referral procedures between 2007 and 2020.

**Method:**

In a comprehensive analysis of the changes made for antibiotics, we extracted information on changes from the European Commission community register of medicinal products and the European Medicines Agency’s database for antibiotics that went through referrals. Changes made in the specific sections of the summary of product characteristics of each referral procedure were scrutinised.

**Results:**

We identified 15 antibiotics from seven classes of antibiotics during the study period. The outcome of all referrals included the restriction of antibiotic use. Therapeutic indications were revised for all antibiotics, with septicaemia and gonorrhoea most common diseases removed. Posology and/or method of administration was updated for all; the majority of referrals included adjustment of dosage for specific populations. Information on contraindication (most regarding hypersensitivity) and information on warnings was amended for all referrals.

**Conclusion:**

Our findings highlight the importance of the regulatory actions. The changes made in the product information aim to ensure appropriate use. Ongoing harmonisation activities are likely to lead to further refinements and restrictions on individual antibiotics in support of rational use. However, further research is required to examine the impact of post-referral label changes on the clinical practice.

## Introduction

Antimicrobial resistance (AMR) is one of the most important challenges in modern clinical practice [1,2]. It is a multifactorial problem that must be tackled from different angles [3]. One of these angles pertains to the domain of medicine regulation. The European Medicines Agency (EMA) together with European Union (EU) countries and the European Commission (EC) has a strategy for AMR that covers also the re-evaluation of the product information that is available to the European patients and healthcare professionals. This process occurs via specific regulatory measures called referral procedures [4].

Although recent advances have been made in propagating prudent antibiotic use through harmonised treatment guidance [5-7], historically, differences in clinical practices exist between EU countries, which is also reflected in differences in the nationally approved product information of antibiotics across Europe. As provided in the EU legislation relating to medicinal products for human use [8], the referral is a post-authorisation procedure that may be initiated under specific conditions such as disagreements among Member States on the use of the medicine or concerns relating to the quality, safety or efficacy of a medicine or a class of medicines. The outcome of a referral may lead to maintenance or regulatory actions such as revocation, suspension or variation to the terms of the marketing authorisations. A variation may cover changes in therapeutic indication, dose recommendations or raising healthcare professionals’ and patients’ awareness regarding particular risks. 

Addressing AMR relies on good antimicrobial stewardship and adherence to clinical treatment guidance. However, up-to-date product information such as a summary of product characteristics (SmPC) based on the available scientific evidence may also be crucial to sustain safe and effective use of the available antibiotics. Within a referral procedure, all available scientific evidence is reviewed. Typically, the outcome of a referral is an amended product information for healthcare professionals and patients reflecting a consensus on product characteristics (i.e. regarding therapeutic indication, dosage, contraindications, adverse events, warning and precautions) that ideally reflects the current state of knowledge and science. The underlying policy strategy is that EU-wide harmonised and adequate instructions for healthcare professionals and patients reduce inappropriate use of antibiotics, thereby contributing to a solution for the AMR emergency.

There are several types of referrals in the European regulatory system. AMR-related concerns are mainly addressed under Article 30 and Article 31 of Directive 2001/83/EC [8]. These procedures may concern a specific medicinal product, a range of medicinal products (all containing the same active substance) or a therapeutic class of medicinal products and can be initiated by the EC, one of the EU Member States or the marketing authorisation holders of the concerned medicinal product. It can be triggered in the following specific cases: Article 30, when Member States adopt divergent decisions concerning the authorisation of medicinal products or to promote harmonisation of authorisations for medicinal products. The re-assessment of a medicinal product under Article 30 can be applied to a medicinal product that is approved for different indications in different Member States. Instead, an Article 31 referral can be triggered where the interest of the EU is involved. The term ‘interest of the Union’ refers particularly to the interests of public health related to medicinal products in the EU in light of quality, safety and efficacy data and related to the free movement of products within the EU [9].

The SmPC is an integral part of a marketing authorisation (license) of any medicinal product [10]. It is a document that describes the properties of a medicine, the officially approved therapeutic indications, information on dosing, safety warnings and directions for appropriate use. It is updated throughout the life cycle of the medicinal product as new data relevant to the benefit and risk become available and/or results of studies that may potentially have an impact on the marketing authorisation. A referral procedure may result in adjustments to the SmPC. The SmPC gives essential information for healthcare professionals on how to prescribe the medicinal product safely and effectively. The package leaflets (PL) that are intended for patients are drawn up in accordance with the SmPC. Changes in the SmPC and the PL as a result of a European referral procedure need to be implemented nationally by the Members States. The aim of the current study was to evaluate such post-authorisation changes made between 2007 and 2020 in the product information of a number of key antibiotics as a result of completed EU referral procedures.

## Methods

### Data source

We performed a comprehensive analysis of the changes made for a number of antibiotics that went through Article 30 and Article 31 referral procedure between 2007 and June 2020. Publicly available information on regulatory procedures and assessment reports from the EC Union register of medicinal products [11] and the EMA database [12] were used to identify products that went through Article 30 and 31 during the study period. The EC Union register contains, among others, the EC decisions with annexes after the referral procedures. For this study, we used Annex II that summarises scientific conclusions and grounds for the variation to the terms of the marketing authorisations. The study includes all antibiotics that were re-assessed between 2007 and 2020 as a part of the European strategy to support the prevention of AMR. 

Referrals that were triggered following safety concerns of the antibiotics were excluded, such as the quinolone- and fluoroquinolone-containing medicinal products referral finalised in November 2018. This procedure was initiated because of serious, disabling and potentially permanent side effects of quinolone and fluoroquinolone antibiotics.

### Changes in the summary of product characteristics following a referral procedure

We analysed in detail the changes made in the SmPCs and the overall outcome of the procedures concerning the benefit-risk balance. A change was defined as any modification made as a result of the referral procedure in the relevant sections of the SmPCs: section 4.1. therapeutic indications, section 4.2. posology (dosage) and method of administration, section 4.3. contraindications and section 4.4. special warnings and precautions for use. For section 4.1., a change was further defined as a number of indications (including prophylactic and non-prophylactic treatment) deleted or added by the European regulators or withdrawn by the marketing authorisation holder; for section 4.2., modifications in posology and method of administration were defined as a number of amendments in posology, adjustments of dosage for specific populations and amendments in the methods of administration; for section 4.3., modifications in contraindication, i.e. harmonisation or deletion, were assessed and for section 4.4., any amendments including harmonisations of warnings were registered.

### Data analysis

We report descriptive data for each antibiotic including the type of antibiotic, the type of referral (Article 30 or 31), the competent authority starting the procedure (EC, Member State) and the period of completing the EC decision (2007–12, 2013–20). The evaluated antibiotics represented seven different classes of antibiotics based on the anatomical therapeutic chemical (ATC) classification system [13]: cephalosporins (ATC J01DC or J01DD), penicillins (ATC J01CA or J01CR), fluoroquinolones (ATC J01MA), carbapenems (J01DH), glycopeptides (ATC J01XA or A07AA), polymyxins (ATC J01XB) and other antibacterial medicines (ATC J01XX).

## Results

### Identification of products

We identified 15 antibiotics for which there had been a referral procedure during the study period 2007 to 2020, representing seven classes of antibiotics (Table 1, Supplementary table S1). Four referrals related to cephalosporins, three to penicillins, two each to fluoroquinolones, carbapenems and glycopeptide and one each to polymyxins and other antibacterial medicines. Most of the regulatory actions (n = 12) were Article 30 Directive 2001/83/EC and only three were Article 31 of Directive 2001/83/EC. Only four referral procedures were triggered by a Member State (France, Germany, the Netherlands and Spain), others (n = 11) were initiated by the EC.

**Table 1:** Product and referral characteristics of antibiotics that went through referral procedures, European Union, 2007–2020 (n = 15)

Type of antibiotics	Article	Competent authority^a^	Year of EC decision
Cephalosporins			
Ceftazidime	30	EC	2011
Cefuroxime axetil	30	EC	2012
Cefuroxime sodium	30	EC	2012
Ceftriaxone	30	EC	2014
Penicillins			
Amoxicillin/clavulanic acid	30	EC	2009
Piperacillin/tazobactam	30	EC	2011
Amoxicillin	30	EC	2015
Fluoroquinolones			
Ciprofloxacin	30	FR	2008
Levofloxacin	30	EC	2012
Carbapenems			
Meropenem	30	EC	2009
Imipenem/cilastatin	30	NL	2011
Glycopeptides			
Teicoplanin	30	EC	2013
Vancomycin	31	ES	2017
Polymyxins			
Colistin/colistimethate sodium	31	EC	2014
Other antibacterial medicines			
Fosfomycin	31	DE	2020

DE: Germany; EC: European Commission; ES: Spain; FR: France; NL: the Netherlands.

^a^ Competent authority starting the procedure.

### Changes in the summary of product characteristics following regulatory referral

A summary of the changes in the SmPC as a result of referral procedures is presented in Table 2. The outcome of all 15 referrals included the restriction of antibiotic use. The amendments such as deletion and addition of an indication per medicinal product in the section 4.1 varied from two for meropenem up to 16 for fosfomycin. The outcomes of 13 referrals included deletion of therapeutic indication that involved non-prophylactic and prophylactic treatment. Only one referral included deletion of both a prophylactic and a non-prophylactic treatment for a single indication (amoxicillin used for endocarditis). Meropenem and colistin/colistimethate sodium were the only antibiotics where a referral did not result in deletion of therapeutic indication, but involved restriction of use. For meropenem there was one addition in the therapeutic indication statement regarding the paediatric population, i.e. 3 months as the lower age limit for treatment of bronchopulmonary infections in cystic fibrosis and complicated urinary tract infections. For colistin/colistimethate sodium the section was revised as follows: colistin/colistimethate sodium are now indicated in adults and children including neonates for the treatment of serious infections with selected aerobic Gram-negative bacteria in patients with limited treatment options. The majority (13/15), of referral procedures resulted in a deletion of at least two therapeutic indications in section 4.1. (Supplementary table S2). Septicaemia was the most common disease removed from the SmPC section 4.1. of the antibiotics included (ciprofloxacin, amoxicillin/clavulanic acid, cefuroxime sodium and teicoplanin), followed by community-acquired pneumonia (ceftazidime, cefuroxime axetil and fosfomycin), gonorrhoea (cefuroxime axetil, cefuroxime sodium, amoxicillin), bone and joint infections (piperacillin/tazobactam, imipenem/cilastatin and cefuroxime sodium) and uncomplicated skin and soft tissue infections (levofloxacin, teicoplanin, ceftriaxone). Meningitis (imipenem/cilastatin and cefuroxime sodium) ranked at an equal rate with urethritis (cefuroxime axetil and amoxicillin), gynaecological infections (piperacillin/tazobactam and cefuroxime sodium), upper respiratory tract infections (cefuroxime sodium and fosfomycin) and lower respiratory tract infections (ceftazidime and imipenem/cilastatin). There were also several diseases or groups of infections that were deleted from section 4.1. for a single referral procedure. For instance the following indications were removed (antibiotics between brackets): sinusitis (ceftriaxone), tonsillitis (amoxicillin/clavulanic acid), asymptomatic bacteriuria and acute cystitis during pregnancy (fosfomycin trometamol oral solution), hospital-acquired pneumonia (levofloxacin), bronchitis (amoxicillin) or *Clostridioides difficile* infection (vancomycin oral route).

**Table 2:** Changes in different sections of the summary of product characteristics of antibiotics as a result of referral procedures, European Union, 2007–2020

Summary of product characteristics section		Referrals
**4.1 Therapeutic indications**		**15**
Addition/revision		2
Deletion		13
Deletion of: group of indication, a disease specific indication, treatment use and prophylactic use		5
Deletion of a disease specific indication, group of indication and treatment use		5
Deletion of a disease specific indication, treatment use and prophylactic use		1
Deletion of a disease specific indication and treatment use		1
Deletion of group of indication and treatment use		1
**4.2 Posology and method of administration**		**15**
Modification in posology		3
Adjustment of dosage for specific population	Paediatric patients	5
	Paediatric patients and patients with reduced renal function	1
	Paediatric patients and patients with reduced renal and hepatic function	1
Changes in the method of administration		1
Changes in the method of administration and adjustment of dosage for patients with reduced renal function		1
Changes in the method of administration and adjustment of dosage for paediatric patients		1
Changes in the method of administration, adjustment of dosage for paediatric patients and patients with reduced renal function		2
**4.3. Contraindications**		**15**
Harmonisation		11
Hypersensitivity		9
Use during pregnancy and in patients with G6PD^a^		1
Hypersensitivity and children under 12 years		1
Deletion		2
Unchanged		2
**4.4. Warnings**		1**5**
Amendment/harmonisation		15

^a^ Patients with glucose-6-phosphate dehydrogenase deficiency.

All referrals resulted in a change in section 4.2. (posology and method of administration). A change in posology was observed for all antibiotics included in this study. For some antibiotics (piperacillin/tazobactam, cefuroxime sodium, ceftriaxone, teicoplanin and colistin/colistimethate sodium), the amendments applied to the method of administration. There were several adjustments of dosage and/or period of treatment for specific populations such as the paediatric population (meropenem, amoxicillin/clavulanic acid, piperacillin/ tazobactam, imipenem/ cilastatin, cefuroxime axetil, teicoplanin, ceftriaxone, amoxicillin, vancomycin, and colistin/ colistimethate sodium, fosfomycin), patients with reduced renal function (amoxicillin/clavulanic acid, cefuroxime sodium, teicoplanin, ceftriaxone, amoxicillin, vancomycin, colistin/colistimethate sodium and fosfomycin) or patients with reduced hepatic function (amoxicillin/clavulanic acid, cefuroxime sodium, ceftriaxone, and colistin/colistimethate sodium).

Among all referral procedures, 11 concluded with a harmonisation of section 4.3. on contraindications. The majority of the harmonised contraindications were regarding information on hypersensitivity (meropenem, amoxicillin/clavulanic acid, piperacillin/tazobactam, ceftazidime, imipenem/cilastatin, cefuroxime axetil, teicoplanin, ceftriaxone, amoxicillin, fosfomycin). One involved harmonisation of levofloxacin with regards to use during pregnancy and in patients with glucose-6-phosphate dehydrogenase (G6PD) deficiency and one concerned oral formulation of fosfomycin in children younger than 12 years. Moreover, two of the 15 referral procedures had a contraindication deleted: for cefuroxime sodium in patients with hepatic dysfunction (lack of evidence) and for colistin/colistimethate sodium-containing products in patients with myasthenia gravis (replacement with a warning in section 4.4). 

The section 4.4 on special warnings and precautions for use was updated/harmonised in all 15 referral procedures. For example, in order to mitigate the well-known risks of using vancomycin, warnings against the potential for systemic absorption, nephrotoxicity, ototoxicity, drug integrations, development of drug-resistant bacteria and recommendations for regular monitoring of renal function were included. For colistin or colistimethate sodium, in addition to myasthenia gravis mentioned above, a warnings advising against concurrent administration of nephrotoxic and/or neurotoxic medicinal products were introduced as well as recommendations to perform regular monitoring of renal function, mentioning the potential correlation with cumulative dose and treatment duration. For fosfomycin, two warnings were included, one regarding the need for combination therapy to reduce the risk of emerging resistance and the second concerning the risk of hypernatraemia and the need to monitor sodium and potassium level because of the risk of sodium overload related to infusion of intravenous fosfomycin.

## Discussion

We have provided an analysis of all antibiotics that were included in the EMA, Member States and EC harmonisation strategy between 2007 and 2020 to address inappropriate use of frequently used, mostly ‘old’, antibiotics. We found that 15 referral procedures were triggered; for all of them a positive benefit–risk balance was confirmed. The therapeutic use was restricted for all included antibiotics. Cephalosporins and penicillins were the two most represented classes of antibiotics in the study. According to the European Centre for Disease Prevention and Control annual report on antimicrobial consumption from 2017, those two classes (together with macrolides and tetracyclines) are among the most consumed of the EU antibacterial medicines for systemic use [14]. Since 2013, the European regulatory network has moved from Article 30 to Article 31 of Directive 2001/83/EC because of, among others, the complexity of engaging with multiple marketing authorisation holders. This has not let to major differences in the outcome of the Article 30 compared with the Article 31.

We assessed changes that were made during the reassessment of product information provided to healthcare professionals and patients. The antibiotic with the highest number of changes following the referral finalised in June 2020 was fosfomycin, where changes applied to all SmPC sections assessed in this study. In total, 17 indications were removed for fosfomycin (including community-acquired pneumonia, acute uncomplicated urinary tract infections in children and periprocedural prophylaxis). It should also be noted that the benefit–risk balance of fosfomycin for intramuscular use and fosfomycin 2 g granules for oral solution was considered negative.

The most notable referral procedure was on colistin/colistimethate sodium. These antibiotics belong to the class of polymyxins that is currently listed among the highest priority critically important antimicrobial medicines [15]. The usage of colistin was minimal because of its nephrotoxicity and neurotoxicity, however, it was reintroduced during the last years as a result of the emergence of multidrug-resistant microorganisms [16]. Several publications in the scientific literature claim that owing to limited therapeutic options against serious infections with Gram-negative bacteria, colistin usage has increased considerably in the last years [17-19]. Therefore, it is crucial that the most up-to-date scientific evidence is included in the SmPC of this class of antimicrobials. During the referral procedure for colistin/colistimethate sodium, the therapeutic indications were restricted to serious infections with selected aerobic Gram-negative bacteria in patients with limited treatment options. This approach is in line with current evidence which shows that polymyxins have assumed an important role as salvage therapy for otherwise untreatable Gram-negative bacterial infections, most notably multidrug-resistant and extensively drug-resistant strains of *Pseudomonas aeruginosa, Acinetobacter baumannii* and Enterobacteriaceae [20]. Several therapeutic indications were deleted in line with the national guidelines. For instance, ceftriaxone should not be used for the treatment of pharyngitis and amoxicillin/clavulanic acid should not be used for tonisillitis [21]. In addition, important changes in SmPC were also made with regard to treatment options for therapeutic indications of respiratory tract infections, including deletion of bronchitis from the amoxicillin SmPC and lower respiratory tract infections from the ceftazidime and imipenem/cilastatin SmPC. According to the literature, acute bronchitis is the most common of lower respiratory tract infections. Despite well-known scientific data that the majority of acute bronchitides in healthy adults are caused by viral infections, antibiotics are still being prescribed [22,23]. Deleting acute bronchitis from therapeutic indications (section 4.1. of the SmPC) for amoxicillin is the first step to decrease usage of these antibiotics. The most common disease removed from the SmPC of four referral procedures (ciprofloxacin, amoxicillin/clavulanic acid, cefuroxime sodium, teicoplanin) was septicaemia. This very complex disease triggers sepsis that causes between 6 and 9 million deaths every year worldwide [24]. Immediate selection of the appropriate antibiotic treatment is essential [25]. Septicaemia was removed from the SmPC mainly owing to insufficient data provided to support this indication. In addition, it was not considered to represent correct therapeutic indication and was replaced with bacteraemia, however we would expect this replacement to have a minimum impact on antimicrobial stewardship.

All referral procedures updated the SmPC with the latest scientific knowledge available at the time of referral on the use of antibiotics for specific population such as children. Not only is it important to select the appropriate antibiotic when treating children, but also to administer the correct dose, taking into account potential differences in pharmacokinetics [26]. Adjustment of dosage for paediatric patients was found in the majority of referral procedures. The European Committee on Antimicrobial Susceptibility Testing (EUCAST) recommends updates on dosing recommendations every year, often increasing the doses [27]. Our current study shows that the vast majority of antibiotics had divergent SmPC at the national level. The overall outcome of regulatory actions ensures that these antibiotics which have been on the market for several decades, continue to be relevant antimicrobial medicines. The harmonisation of information available to EU healthcare professionals and citizens is an important step in ensuring an appropriate use of antibiotics and at the same time addressing AMR. The SmPCs provide up-to-date scientific knowledge that should encourage rational and sustainable use of antibiotics. Whether clinical practice will adopt and implement the new updated evidence-based product information and enforce these recommendations in daily clinical practice remains to be evaluated. Restriction of use or posology adjustment may also have yet unforeseen consequences to clinical practice. The impact of post-referral prescribing trends for various medicines has been assessed in a study on the impact of the EU label changes for systemic diclofenac after referral procedure [28]. We acknowledge that the impact of referral procedures is likely to differ between primary and secondary care. Our future research will aim to look closer at the impact of the regulatory changes described here on antimicrobial clinical practice.

To our knowledge, our study was the first to provide a comprehensive analysis of the changes made during the referral procedures on antibiotics.

## Conclusion

Our findings highlight the importance of the European regulatory actions, i.e. referral procedures over the last decade, on updating product information of key antibiotics as laid down in the SmPC and PL. The majority of the referrals’ outcomes can be linked to restriction of use (i.e. the indication) and therefore can be considered as an important step against AMR. Inappropriate and unjustified use of antibiotics remains a critical determinant of AMR. Further studies on antibiotic utilisation, clinical practice changes and AMR epidemiology should provide more insight into the real public health impact, both positive and negative, of these regulatory measures. We expect more ongoing harmonisation activities from the EU regulatory network in order to restrict the use of antibiotics further. Therefore, further research is needed to understand better the relationship between the regulatory actions and antimicrobial use, which will be the focus of our future work.

## Supplementary Data

695000 Supplement
