# Supplementary material for: Enabling appropriate use of antibiotics: review of European Union procedures of harmonising product information, 2007 to 2020
Source: Euro Surveill. 2020 Nov 12;25(45):2000035. doi: 10.2807/1560-7917.ES.2020.25.45.2000035 (PMC7667629; doi:10.2807/1560-7917.ES.2020.25.45.2000035)
Supplement: Supplement [file 20-00035_OPALSKA_Supplement.pdf]

This supplementary material is hosted by *Eurosurveillance* as supporting information alongside the article *Enabling appropriate use of antibiotics: review of European Union procedures of harmonising product information, 2007 to 2020*, on behalf of the authors, who remain responsible for the accuracy and appropriateness of the content. The same standards for ethics, copyright, attributions and permissions as for the article apply. Supplements are not edited by *Eurosurveillance* and the journal is not responsible for the maintenance of any links or email addresses provided therein."

Supplementary Table S1. Product and referral characteristics of antibiotics that went through referral procedures, European Union, 2007–2020 (n = 15)

Class of antibiotics	Medicinal product	International nonproprietary name	ATC code	Year[b]	Article	Competent authority[a]	EC decision
Cephalosporins							
	Fortum	Ceftazidime	J01DD02	1983	30	EC	13.01.2011
	Zinnat	Cefuroxime axetil	J01DC02	1987	30	EC	23.08.2012
	Zinacef	Cefuroxime sodium	J01DC02	1987	30	EC	10.09.2012
	Rocephin	Ceftriaxone	J01DD04	1982	30	EC	21.03.2014
Penicillins							
	Augmentin	Amoxicillin/ clavulanic acid	J01CR02	1972	30	EC	18.10.2009
	Tazocin	Piperacillin/ tazobactam	J01CR05	1992	30	EC	21.02.2011
	Amoxil	Amoxicillin	J01CA04	1972	30	EC	20.08.2015
Fluoroquinolones							
	Ciproxin	Ciprofloxacin	J01MA02	1987	30	FR	07.10.2008
	Tavanic	Levofloxacin	J01MA12	1993	30	EC	31.07.2012
Carbapenems							
	Meronem	Meropenem	J01DH02	1994	30	EC	15.10.2009
	Tienam	Imipenem/ cilastatin	J01DH51	1984	30	NL	10.03.2011
Glycopeptides							
	Targocid	Teicoplanin	J01XA02	1986	30	EC	12.09.2013
	Vancomycin-containing medicines	Vancomycin	J01XA01(IV) A07AA09 (PO)	1955	31	ES	21.09.2017
Polymyxins							
	Polymyxin-based products	Colistin/ colistimethate sodium	J01XB01	1960	31	EC	16.12.2014
Other antibacterial medicines							
	Fosfomycin Containing medicines	Fosfomycin trometamol/ Fosfomycin calcium/ Fosfomycin disodium/ Fosfomycin sodium	J01XX01	1969	31	DE	09.06.2020

DE: Germany; EC: European Commission; ES: Spain; FR: France; NL: the Netherlands.

[a] Competent authority starting the procedure.

[b] Year of the INN authorisation.

Supplementary Table S2 - Therapeutic indications removed from the section 4.1 of the summary of product characteristics of antibiotics as a result of referral procedures, European Union, 2007–2020

**Removed therapeutic indications ***	**Medicinal product**<br>**All formulation unless specified next to an antibiotic**<br>**(IV- intravenous, PO – per oral, OS – oral solution)**
Disease treatment (unless specify prophylaxis):	
Abundant asymptomatic bacteriuria	fosfomycin trometamol OS
Acute cholangitis	amoxicillin
Acute bacterial urethrovesical syndrome	fosfomycin trometamol OS
Acute lung disease	amoxicillin
Acute sinusitis	ciprofloxacin
Asymptomatic bacteriuria and acute cystitis during pregnancy	fosfomycin trometamol OS
Bronchitis	amoxicillin
Cholecystitis	amoxicillin
Clostridioides difficile infection (prophylaxis)	vancomycin PO
Community-acquired pneumonia	ceftazidime
	cefuroxime axetil
	fosfomycin IV
Decontamination of the gastrointestinal tract in immune-compromised patients when combined with an aminoglycoside	vancomycin PO
Endocarditis	imipenem/ cilastatin
Endocarditis (treatment and prophylaxis)	amoxicillin PO
Enteritis with bacteraemia	amoxicillin
Gonococcal arthritis	ceftriaxone
Gonococcal eye infection	ceftriaxone
Gonococcal infection	amoxicillin
Gonorrhoea	cefuroxime axetil
	cefuroxime sodium
	amoxicillin
Hospital-acquired pneumonia	levofloxacin
Meningitis	imipenem/ cilastatin
	cefuroxime sodium
Non-specific urethritis	fosfomycin trometamol OS
Peritonitis	amoxicillin
Pharyngitis	ceftriaxone
Prostatitis	ceftriaxone
Purpura fulminans	ceftriaxone
Selective digestive decontamination in immuo-suppressed patients	ciprofloxacin
Septicaemia	ciprofloxacin
	amoxicillin/ clavulanic acid
	cefuroxime sodium
	teicoplanin
Sinusitis indication	ceftriaxone
Staphylococcal enterocolitis	vancomycin PO
Tonsillitis	amoxicillin/ clavulanic acid
Urethritis	cefuroxime axetil
	amoxicillin
Ventilator associated pneumonia	ceftazidime
Group of infections treatment (unless specify prophylaxis):	
Acute uncomplicated urinary tract infections in children	fosfomycin trometamol OS
	fosfomycin calcium PO
Bone infections	piperacillin/ tazobactam
	Imipenem/ cilastatin
	cefuroxime sodium

Dental surgery (prophylaxis)	teicoplanin
Dermatological infections	fosfomycin calcium PO
Female genital infections	amoxicillin (parenteral formulation)
Gynaecological infections	piperacillin/ tazobactam
	cefuroxime sodium
Gastrointestinal infections	fosfomycin calcium PO
Gastrointestinal infections	fosfomycin calcium PO
Indications based on fosfomycin´s antibacterial activity and pharmacokinetic properties; Indications restricted to severe infections caused by microorganisms defined as susceptible in pharmacodynamics and Methicillin-resistant staphylococcal meningitis	fosfomycin IV
Joint infections	piperacillin/ tazobactam
	imipenem/ cilastatin
	cefuroxime sodium
Lower respiratory tract infections	ceftazidime
	imipenem/ cilastatin
Male genital infections	amoxicillin
Mixed infections	imipenem/ cilastatin
Obstetric infections	cefuroxime sodium
Ophthalmological infections	fosfomycin IV
Orthopaedic surgery (prophylaxis)	teicoplanin
Peri-operative infections	fosfomycin IV
Periprocedural (prophylaxis)	fosfomycin trometamol OS
Post-surgical infections (prophylaxis)	imipenem/ cilastatin
Post-operative treatment of urinary tract infections	fosfomycin trometamol OS
Prophylaxis of invasive infections due to *Neisseria meningitides* in adults	ciprofloxacin IV
Recurrent urinary tract infections	fosfomycin trometamol OS
Respiratory tract infection	ceftazidime
Serious infections caused by *Haemophilus Influenzae*	amoxicillin
Severe infections of other organ systems due to fosfomycin-susceptible Gram-negative pathogens with limited therapeutic options	fosfomycin IV
Uncomplicated skin and soft tissue infections	levofloxacin
	teicoplanin
	ceftriaxone
Uncomplicated urinary tract infections	ceftriaxone
Upper respiratory tract infections	cefuroxime sodium
	fosfomycin IV

* References to the detailed information for each antibiotic:

Amoxicillin - https://www.ema.europa.eu/en/medicines/human/referrals/amoxil
Amoxicillin/ clavulanic acid - https://www.ema.europa.eu/en/medicines/human/referrals/augmentin
Ceftazidime - https://www.ema.europa.eu/en/medicines/human/referrals/fortum
Ceftriaxone - https://www.ema.europa.eu/en/medicines/human/referrals/rocephin
Cefuroxime axetil - https://www.ema.europa.eu/en/medicines/human/referrals/zinnat
Cefuroxime sodium - https://www.ema.europa.eu/en/medicines/human/referrals/zinacef
Ciprofloxacin - https://www.ema.europa.eu/en/medicines/human/referrals/ciprofloxacin-bayer
Colistin/ Colistimethate sodium - https://www.ema.europa.eu/en/medicines/human/referrals/polymyxin-containing-medicines
Fosfomycin/ fosfomycin trometamol & fosfomycin calcium - https://www.ema.europa.eu/en/medicines/human/referrals/fosfomycin-containing-medicinal-products
Imipenem/ cilastatin - https://www.ema.europa.eu/en/medicines/human/referrals/tienam
Levofloxacin - https://www.ema.europa.eu/en/medicines/human/referrals/tavanic
Meropenem - https://www.ema.europa.eu/en/medicines/human/referrals/meronem
Piperacillin/ tazobactam - https://www.ema.europa.eu/en/medicines/human/referrals/tazocin
Teicoplanin - https://www.ema.europa.eu/en/medicines/human/referrals/targocid-associated-names
Vancomycin - https://www.ema.europa.eu/en/medicines/human/referrals/vancomycin-containing-medicines
